# New mutation in WT1 gene in a boy with an incomplete form of Denys-Drash syndrome: A CARE-compliant case report

**DOI:** 10.1097/MD.0000000000025864

**Published:** 2021-05-14

**Authors:** Nail R. Akramov, Rafael F. Shavaliev, Ilsiya V. Osipova

**Affiliations:** a Kazan State Medical University; b Republican Clinical Hospital of the Ministry of Health of the Republic of Tatarstan; c Children's Republican Clinical Hospital of the Ministry of Health of the Republic of Tatarstan, Kazan, Russian Federation.

**Keywords:** children, Denys-Drash syndrome, disorder of sexual development 46, hypospadias, surgical treatment, Wilms’ tumor, WT1 gene mutation, XY

## Abstract

**Rationale:**

Pediatric patients with WTl-associated syndromes (including Wilms’ tumor-aniridia syndrome and Denys-Drash syndrome), Perlman syndrome, mosaic aneuploidy, and Fanconi anemia with a biallelic breast cancer type 2 susceptibility protein mutation have the highest risk of developing Wilms’ tumor.

**Patient concerns and diagnosis:**

We describe a patient with bilateral metachronous Wilms’ tumor, ambiguous genitalia characterized by 46, XY disorder of sexual development (DSD) with scrotal hypospadias and bilateral abdominal cryptorchidism, but without nephropathy. At the age of 7 months, the child underwent left nephrectomy with left orchiopexy. At follow-up after 8 months, a second tumor with a diameter of 10 mm was detected in abdominal CT scans at the lower pole of the right kidney.

**Intervention:**

Intra-operative macroscopic inspection of the right kidney revealed a tight attachment of the right proximal ureter to the tumor. Thus, retroperitoneoscopic resection of the lower pole of the right kidney had to be changed to an open surgical procedure with partial resection of the proximal ureter and high uretero-ureterostomy. We subsequently performed orchiopexy and two-stage correction of hypospadias using a free skin graft.

**Outcomes:**

At the last follow-up at the age of 8 years, no pathology requiring treatment was noted. A pair-end-reading (2 × 125) DNA analysis with an average coverage of at least 70 to 100 × revealed a previously unknown heterozygous mutation in exon 7 of the Wilms’ tumor suppressor gene 1 (WT1) gene (chr11:32417947G>A), leading to the appearance of a site of premature translation termination in codon 369 (p.Arg369Ter, NM_024426.4). This mutation had not been registered previously in the control samples “1000 genomes,” Exome Sequencing Project 6500, and the Exome Aggregation Consortium. Thus, to the best of our knowledge this represents a newly identified mutation causing incomplete Denys-Drash syndrome.

## 1 Introduction

Since the publication of the monograph “Mischgeschwülste der Niere” by Max Wilms in 1899, the most common solid neoplasm of the urinary tract described in children has been Wilms tumor (WT), with an incidence of 3.9 to 10.9 cases per 1 million children.^[1,2]^ WT is the most frequently observed childhood tumor in Europe and North America.^[3]^ The malignant tissue of WT is composed of embryonal tissue consisting of blastemic, epithelial, and stromal components.^[4]^ Overall, 9% of WTs occur in children below the age of 1 year and rank 4th among pediatric tumors in this age group. In 6% to 10% of patients, bilateral disease is found.^[5,6]^

Overall, 75% of WTs occur in children below the age of 5 years.^[7]^ Protocols for the comprehensive management of patients with WT (International Society of Pediatric Oncology, National Wilms’ Tumor Study Group) have been established successfully. If this type of pediatric cancer disease is detected at an early stage, oncologic treatment results are promising.^[8,9]^ Overall survival of children suffering from WT is approximately 80% with a significantly lower survival of children with bilateral tumors.^[10]^ Approximately 10% of WTs are associated with germline mutations and congenital anomalies.^[4]^

In the etiology of this type of childhood cancer, a genetic disposition was identified for roughly 1 third of children affected by WT.^[4,11,12]^ In affected children, mutation of a gene located on the short arm of chromosome 11 at the 13th locus was identified initially.^[13]^ Subsequently, other mutations were identified.^[4,10,12,14]^ Over time, cases of a combination of WT and other diseases have been described, which together give rise to a more severe course of disease.^[4,15]^ WT progression has been linked to impaired expression of genes other than Wilms’ tumor suppressor gene 1 (WT1), WTx, and CTNNB1, which were found in about 1/3 of WT patients.^[11]^ Especially mutations in TP53 appear to be linked to unfavorable histology of WT.^[16,17]^ All of them had similar genetic manifestations, that is, mutation of the WT1 gene (OMIM no. 607102). In 1967 and 1970, one of these syndromes, that is, Denys-Drash syndrome (DDS, OMIM no. 194080) named after its discoverers, was described for the first time. DDS is characterized by a triad of symptoms, that is, nephropathy, 46, XY disorder of sexual development (DSD), and a predisposition to develop WT.^[18,19]^

DDS patients with XY karyotype may suffer from a spectrum of DSD disorders.^[20]^ More than 90% of DDS patients carry heterozygous de novo germ line WT1 mutations located at chromosome 11p13. The WT1 gene comprises 10 exons encoding a zinc-finger transcription factor required for development of kidney and gonads. Hereby, exons 1 to 6 encode the N-terminal transactivation domain.^[21,22]^ Exons 7 to 10 encode the C-terminal DNA binding domain with 4 zinc fingers.^[21,22]^

Most DDS patients present with missense mutations in the C-terminal DNA binding domain affecting most frequently the hot-spot exon 8 and 9 encoding the zinc fingers 2 and 3.^[23,24]^ The mutant protein thereby loses the DNA-binding ability although it retains the N-terminal self-association domain required for dimerization.^[25]^ Mutations in the N-terminal region most frequently present nonsense mutations or frame shift mutations. These produce truncated proteins lacking the regions required for WT1 self-association, DNA-binding activities, and transactivation.^[26]^

This case report describes the study of the gene mutation underlying DDS as well as management and outcome of the illness in a 9-year old child.

## 2 Case presentation

We obtained written informed consent from the parents of our patient before reporting on this child. The patient was born on December 1, 2011, as the second child of a family (the first boy had no urologic pathology). No consanguinity was reported for the parents of this patient. After birth, the extragenital pathology indicated DSD in the form of bilateral cryptorchidism and scrotal hypospadias. The child was scheduled for surgical treatment at the end of the first year of life. However, at the age of 6 months, his mother discovered a tumoros formation in the abdomen while bathing the baby. An ultrasound scan was performed at the outpatient clinic of the local hospital, and a tumor of the retroperitoneum was diagnosed.

**Figure 1:**
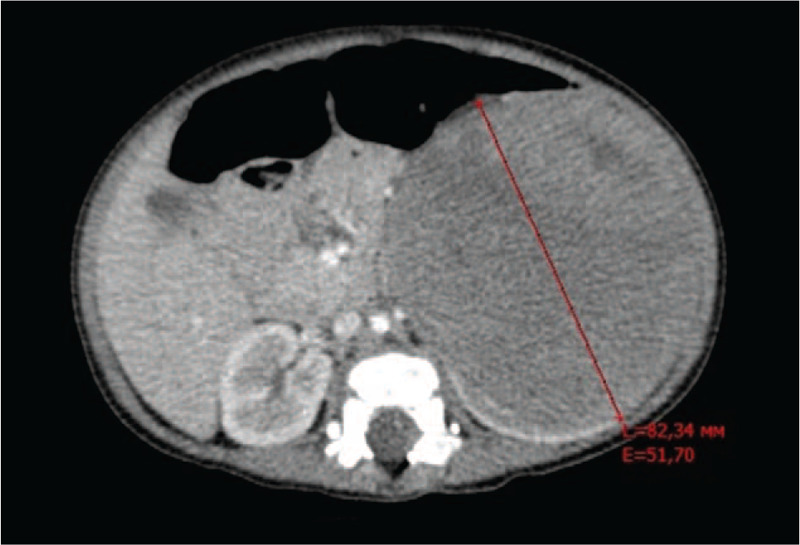
Contrast-enhanced CT scan obtained at the patient's age of 6 months. A large, solid, expansive tumor replaced the left kidney. There was no enlargement of lymph nodes, and the right kidney showed no tumor signs.

The child was referred to our hospital for further evaluation and treatment. Abdominal contrast-enhanced computed tomography (CT) was performed. This revealed a large tumor of the left kidney, combined with malformations of the genitourinary system (Fig. 1). The results of the examination were consistent with the diagnosis of nephroblastoma of the left kidney T3 NH M0. Bilateral abdominal cryptorchidism was confirmed. When the boy was 7 months old, we removed the tumor of the left kidney by open tumor nephrectomy and performed orchiopexy of the left undescended testis. The child showed no dysmorphic signs associated with DSD, and ophthalmologic, neurologic, and psychiatric evaluations showed normal findings.

The mass of the removed tumor and kidney weighed 520 g, accounting for about 10% of the body weight of the child. Histopathologic examination of the tumor revealed “mixed nephroblastoma,” and TNM classification was pT3 N0 M0. The postoperative course was uneventful, and the members of our pediatric oncologic tumor board decided not to conduct postoperative chemotherapy.

**Figure 2:**
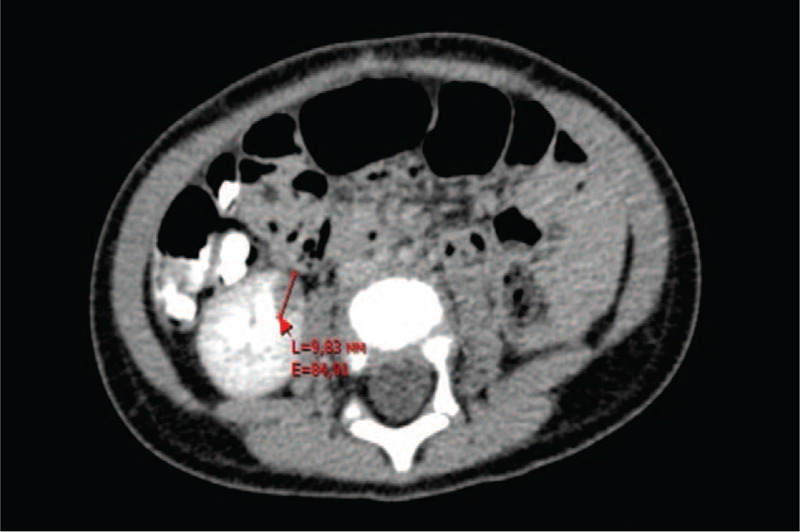
Contrast-enhanced CT scan obtained 8 months after tumor nephrectomy of left kidney showing an oval hypodense lesion at the medial aspect of the right kidney with a maximum diameter of 10 mm.

At follow-up after 8 months, a spherical lesion (diameter: 10 mm) at the lower pole of the right kidney was detected in abdominal CT scans (Fig. 2). In view of this new finding, the initial diagnosis was corrected to bilateral metachronic nephroblastoma pT4 NH M0.

We opted for retroperitoneoscopy to resect the lower pole of the right kidney, but because of macroscopic involvement of the proximal right ureter, we switched to open minilumbotomy for resection of the lower pole of the right kidney, partial resection of proximal right ureter, and uretero-ureterostomy with insertion of a ureteral stent. Histopathologic investigation of the kidney tumor on the left side revealed mixed nephroblastoma (epithelial-blastemic) G2 with tumor-free resection margins (R0). Histopathologic examination of the tumor of the right lower pole showed cystic nephroma (G1). Resection margins were free of tumor tissue (R0). Histopathologic examination of the right proximal ureter confirmed that the ureteric wall was not invaded by the tumor.

After 2 months, we removed the ureteral stent by cystoscopy. At follow-up after 1 year, the child was free of pain and exhibited adequate body height and weight gain for age. CT scan confirmed the absence of recurrent nephroblastoma. We undertook orchiopexy to correct cryptorchidism on the right side when the child was 2.5 years old. After 8 months, we undertook Bracka-I urethroplasty, in which a free skin graft of the foreskin was used. There were no postoperative complications, and the graft healed uneventfully. We performed tubularizing urethroplasty 10 months after Bracka I procedure.^[27]^

**Figure 3:**
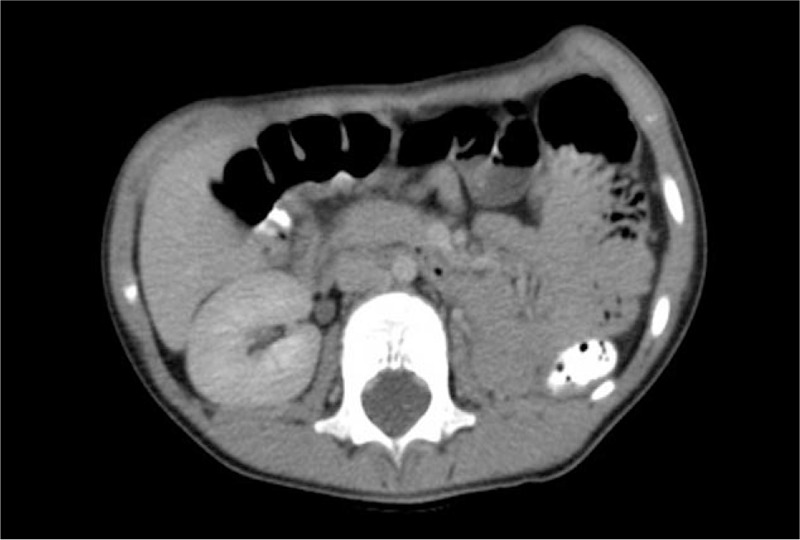
CT scan obtained at the patient's age of 4 years and 10 months. The right kidney appeared normal after partial resection of the lower pole. There was no dilatation of the right renal pelvis after high uretero-ureterostomy.

**Figure 4:**
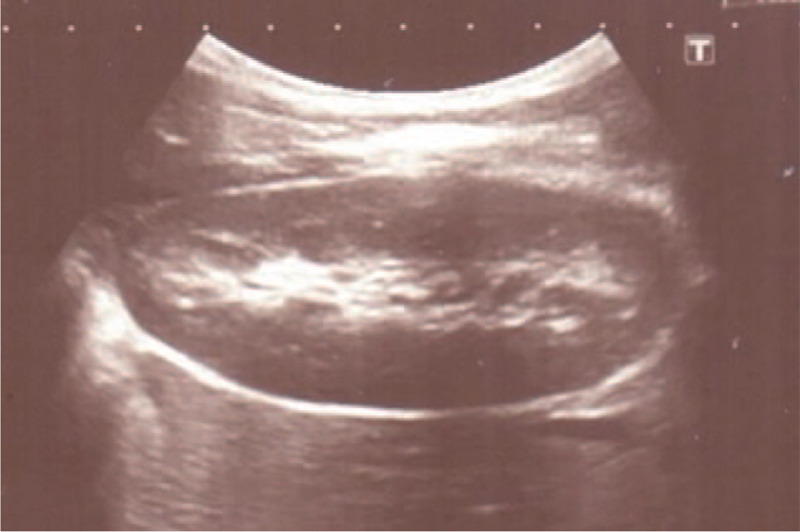
Renal ultrasonography obtained at the patient's age of 7 years and 3 months showing normal structure of parenchyma of the right kidney and no dilation of the renal pelvis.

Figure 3 shows a CT scan obtained 3.5 years after resection of the lower pole of the right kidney. No residual tumor was noted and complete remission of nephroblastoma was confirmed. Every subsequent year, the child underwent follow-up examinations with clinical evaluation and ultrasound scans of the abdomen, retroperitoneum, and testes at our institution. At ultrasound examination of the right kidney 6 years after resection of the lower pole of the right kidney, we noted hypertrophy of the remaining right kidney (Fig. 4). No abdominal or retroperitoneal tumor residuals were discernable at ultrasonography. There was no evidence of proteinuria. Levels of urea and creatinine were within the physiologic ranges. The external genitalia appeared normal with a penis of normal size for age. The meatus was located at the tip of the glans, and voiding was described as uneventful with a single stream. Both gonads were residing in the scrotum. The left testicle showed normal size for age, and the right testicle was hypoplastic.

At the last follow-up of the boy at the age of 8.5 years, we recorded a stature-for-age percentile of 35 and a weight-for-age percentile of 3. Urine analysis revealed no proteinuria. The child and his family did not report any urologic complaints or health problems related to DDS or DSD.

After obtaining informed consent from the family, we searched for pathogenic mutations associated with hereditary kidney disease, as well as with other hereditary diseases with similar phenotypic manifestations. DNA analysis was performed by the pair-terminal reading method (2 × 125) with an average coverage of at least 70 to 100 × . For sample preparation, we used the technique of selective capture of DNA regions belonging to the coding regions of genes with known clinical significance. Sequencing data were processed by an automated algorithm. This included alignment of reads to the reference sequence of the human genome (hg19), postprocessing alignments, identifying variants and filtered variants, and annotation of identified variants in all known transcripts of each gene from the RefSeq database using a number of methods for prediction of pathogenicity of substitutions (SIFT, PolyPhen2-HDIV, PolyPhen2 HVAR, MutationTaster, LRT), as well as methods of calculating evolutionary conservative positions (PhyloP, PhastCons). Samples of the 1000 genomes, Exome Sequencing Project 6500, and Exome Aggregation Consortium projects were used to estimate the population frequencies of the identified variants.

We found a previously undefined heterozygous mutation in exon 7 of the WT1 gene (chr11: 32417947G> A), leading to the appearance of a premature translation termination site in the 369 codon (p.Arg369Ter, NM_024426.4). Heterozygous mutations in the WT1 gene leading to impaired synthesis of the full-sized protein have previously been described, particularly in patients with DDS (OMIM: 194080) and WT type 1 (OMIM: 194070). The mutation was not registered in the control samples “1000 genomes,” Exome Sequencing Project 6500, and the Exome Aggregation Consortium. Since the mutation disrupts the synthesis of a full-size protein, it must be considered pathogenic.

## 3 Discussion

DDS is characterized by a variable combination of early onset steroid-resistant nephrotic syndrome, WT, DSD, and gonadoblastoma.^[28]^ There is a paucity of publications of DDS cases, and the classical triad of clinical manifestations appears incomplete in many patients.

The WT1 gene encodes a zinc-finger protein crucial for regulations of many genes by DNA binding.^[29]^ Normal development of gonads, kidneys, and development of the urogenital tract are regulated by WT1.^[16]^ Although we noted a favorable outcome in our patient at long-term follow-up of WT, it must be kept in mind that Perlman et al^[30]^ warned that loss of heterozygosity (LOS) on 11p in children younger than 2 years suffering from small WT and allele loss on 11p puts these children at a greater risk for relapse when treated with minimal (chemonaive) therapy.^[30]^ An investigation of 63 families with 2 or more family members suffering from WT revealed that WT1 mutations account for 6% of familial WT.^[1]^ Cresswell et al demonstrated evidence for 2 separate tumor sites in unilateral WT disease with divergent histology, and in bilateral WT.^[15]^ We observed a bilateral metachronous nephroblastoma formation in our patient characterized by an exon 7 mutation. Cresswell et al showed that bilateral WT appear genetically distinct and probably arise independently.^[15]^ Thus, multiple tumor biopsies are required to assess genetic heterozygosity of WT.^[15]^

The majority of WT1 mutations in DDS patients occurs sporadically and represents missense mutations in exons 8 and 9 of the zinc-finger DNA binding region.^[25]^ In clinical manifestations of DDS, mutations in other initial exons of the 11p13 locus are less common.^[20,31,32]^

We detected a new mutation in exon 7 encoding the first zinc-finger protein. Bruening et al described a case of a mutation in exon 7 in a female patient with nephropathy, but without malignant tumor or DSD.^[33]^ In another study published in 2003, Auber et al described several patients with mutations in the WT1 gene not located at the hot-spot mutation regions at exons 8 and 9 but at exons 3, 4, and 7.^[13]^ In a patient with changes in exon 7, a point mutation was detected, which led to the replacement of arginine 301 with the formation of a stop codon in a patient with karyotype 46, XY. Clinically, DDS was characterized by bilateral inguinal cryptorchidism (testicular hypoplasia was observed at the age of 19 months and testicular insufficiency at the age of 16 years). External genitalia appeared female, with formation of a vagina. However, the uterus was absent. In this patient, bilateral WT was treated with unilateral nephrectomy and resection of the contralateral kidney tumor. In this patient, no nephrotic syndrome occurred.^[13]^

Takata et al also described 2 patients with a mutation in exon 7.^[34]^ A male patient (46, XY) suffered from nephrotic syndrome at the age of 2 years with rapid progression to renal failure but did not have either WT or DSD. The second patient exhibited a similar mutation (46, XY) with clinical manifestations of nephropathy, female genitalia, and DSD, but without WT.^[34]^

In the literature, there are other descriptions of mutations of the WT1 gene protein in uncharacteristic exons, but they have similar manifestation of DDS.^[16,35–37]^ Various mutations of the WT1 gene with atypical clinical manifestations have been grouped into a category termed “non-complete DDS” as proposed by Bardeesy et al in 1994.^[38]^ The authors suggested using this term to describe patients with nephropathy, DSD, or WT.^[34,38]^ The key manifestation of this disease is nephrotic syndrome resistant to pharmacologic treatment, which necessitates kidney transplantation in affected patients.^[34,38]^

Because 46, XY DSD can be associated with variable clinical findings, exome sequencing has been recommended to discover a genetic cause without preconceived phenotype suggestion.^[39]^ Additional investigation of parental samples represents an effective test not only to test for a familiar inherited genetic disorder, but also to determine the pathogenicity of these mutations.^[39]^

In multicenter studies, cases of nephropathy with DSD and/or WT were analyzed.^[16,33–35]^ In all patients, the leading symptom was nephrotic syndrome. Weaver et al at the Washington Children's Hospital introduced a classic DDS case.^[40]^ Their patient with karyotype 46, XY was diagnosed with gonadal dysgenesis, end-stage renal disease, and bilateral WT. Because of the genetically confirmed DDS (mutation in exon 8 in zone 2 of the zinc finger of the WT1 gene), bilateral nephrectomy and bilateral gonadectomy were performed.^[40]^ According to other authors, nephrotic syndrome may appear later or will not manifest itself at all. Nevertheless, this is possible only with mutations in noncharacteristic exons. For example, specialists from the Denver Children's Hospital described a case of mutation in exon 6 of WT1 in a patient with karyotype 46, XY, incomplete androgen insensitivity syndrome, and bilateral WT without nephrotic syndrome.^[41]^ A group of authors from Germany also noted the absence of nephropathy.^[42]^ Among 53 patients included in their study, 3 did not suffer from proteinuria. Two patients with karyotype 46, XY had mutations in exon 1 and bilateral WT. One patient exhibited a mutation in exon 2, and this patient showed 46, XY DSD without nephroblastoma.^[42]^

Baxter et al recommended early identification of the genetic cause of DSD in order to facilitate correct management of the patient by obtaining more focused endocrine and imaging studies. This approach also allows for correct surgical management of DSD patients.^[39]^ Dattolo et al described a patient with a mutation in exon 6 with untypical manifestations.^[43]^ After detecting unilateral WT, nephrectomy was performed. Subsequently, an expansive lesion in the contralateral kidney was identified, but renal dysplasia was confirmed histopathologically. At the age of 15 years, the patient experienced symptoms of nephropathy that increased over time. Hemodialysis was required, and kidney transplantation was performed subsequently. This case represents a variant of untypical manifestation of DDS, even in its incomplete and cross-sectional forms. This underlines the need for a genetic study in patients with WT and nephropathy.^[40]^ Testing for WT1 mutations or deletions is recommended also in 46, XY dysgenesis with structural renal alterations and/or proteinuria.^[44]^

Many authors described unusual situations in patients with presumed pathology associated with the WT1 gene. Finken et al^[45]^ proposed revising the hypothesis of Köhler et al^[44]^ and recommended to conduct genetic studies in all patients with gonadal dysgenesis even if no kidney pathology is evident. Thus, not only patients with 46, XY gonadal dysgenesis with structural changes of the kidneys and/or proteinuria should undergo genetic studies.

Thus, mutations in exons 8 and 9 are always associated with nephropathy. Mutations in other WT1 sites, most often outside the “zinc fingers” zone, can occur without impaired renal function. The cases described above indicate that nephropathy can be absent or appear later in life. This raises the question whether there is a connection between unilateral nephrectomy and kidney resection for the manifestation of nephrotic syndrome. It is unlikely that nephropathy occurs later if it was not encoded by genetic mutation, but an influence of kidney resections on the timing of its manifestation may be responsible for the different time intervals until manifestation of nephrotic syndrome.

The clinical manifestations of DDS differ markedly and require variable treatment. Thus, it may be more reasonable to treat each of these mutations in a patient-specific manner. In DDS patients, proteinuria has been found to occur very early in life. Diffuse mesangial sclerosis or mesangial hyperplasia cause end-stage renal disease in patients suffering from missense point mutations at exons 8 and 9 encoding the 2nd and 3rd zinc-finger region of the protein.^[16]^ In our patient, the point mutation at exon 7 did not result in nephrotic syndrome. This is in accordance with the findings of Auber et al.^[13]^ However, Bruening et al reported on 1 patient with exon 7 mutation suffering from end-stage renal disease.^[33]^

## Acknowledgment

We thank Johannes M. Mayr, MD, PhD, and Silvia M. Rogers, PhD, for revising the text of the manuscript. Johannes M. Mayr, MD, PhD, and Silvia M. Rogers, PhD give permission to be named.

## Author contributions

**Conceptualization:** Nail Ramilovich Akramov.

**Data curation:** Nail Ramilovich Akramov, Ilsiya V. Osipova.

**Formal analysis:** Nail Ramilovich Akramov, Rafael F. Shavaliev, Ilsiya V. Osipova.

**Investigation:** Nail Ramilovich Akramov.

**Methodology:** Nail Ramilovich Akramov, Rafael F. Shavaliev.

**Project administration:** Nail Ramilovich Akramov.

**Visualization:** Nail Ramilovich Akramov, Ilsiya V. Osipova.

**Writing – original draft:** Nail Ramilovich Akramov, Rafael F. Shavaliev, Ilsiya V. Osipova.

**Writing – review & editing:** Nail Ramilovich Akramov, Rafael F. Shavaliev, Ilsiya V. Osipova.
